# Investigation of the Structural, Electrical, and Optical Properties of the Nano-Scale GZO Thin Films on Glass and Flexible Polyimide Substrates

**DOI:** 10.3390/nano6050088

**Published:** 2016-05-10

**Authors:** Fang-Hsing Wang, Kun-Neng Chen, Chao-Ming Hsu, Min-Chu Liu, Cheng-Fu Yang

**Affiliations:** 1 Department of Electrical Engineering and Graduate Institute of Optoelectronic Engineering, National Chung Hsing University, Taichung 402, Taiwan; fansen@dragon.nchu.edu.tw (F.-H.W.); stmike18@yahoo.com.tw (M.-C.L.); 2 Department of Electrical Engineering, Kun-Shan University, Tainan 710, Taiwan; knchen@mail.ksu.edu.tw; 3 Department of Mechanical Engineering, National Kaohsiung University of Applied Science, Kaohsiung 807, Taiwan; jammy@kuas.edu.tw; 4 Department of Chemical and Materials Engineering, National University of Kaohsiung, Kaohsiung 81141, Taiwan

**Keywords:** Ga_2_O_3_-doped ZnO (GZO) thin film, glass, polyimide (PI), X-ray photoelectron spectroscopy (XPS)

## Abstract

In this study, Ga_2_O_3_-doped ZnO (GZO) thin films were deposited on glass and flexible polyimide (PI) substrates at room temperature (300 K), 373 K, and 473 K by the radio frequency (RF) magnetron sputtering method. After finding the deposition rate, all the GZO thin films with a nano-scale thickness of about 150 ± 10 nm were controlled by the deposition time. X-ray diffraction patterns indicated that the GZO thin films were not amorphous and all exhibited the (002) peak, and field emission scanning electron microscopy showed that only nano-scale particles were observed. The dependences of the structural, electrical, and optical properties of the GZO thin films on different deposition temperatures and substrates were investigated. X-ray photoemission spectroscopy (XPS) was used to measure the elemental composition at the chemical and electronic states of the GZO thin films deposited on different substrates, which could be used to clarify the mechanism of difference in electrical properties of the GZO thin films. In this study, the XPS binding energy spectra of Ga2p_3/2_ and Ga2p_1/2_ peaks, Zn2p_3/2_ and Zn2p_1/2_ peaks, the Ga3d peak, and O_1__s_ peaks for GZO thin films on glass and PI substrates were well compared.

## 1. Introduction

Recent advances in nanotechnology have contributed to the development of photon upconversion materials as promising new-generation candidates of fluorescent bioprobes and spectral converters for biomedical and optoelectronic applications [1]. Also, dimension-reduced process to make them approaching two-dimensional nanostructures into precisely controlled lamellar or called layer nanomaterials are currently achievable. In particular, layer-by-layer (LbL) assembly is known as a highly versatile method for fabrication of controlled layered structures from various kinds of component materials [2]. Zhuang *et al.* published a review focusing on networks‘ growth processes of metal-organic frameworks and crystalline coordination from the view of surface chemistry [3]. They mainly focused on the methods involving liquid phases, and those methods can be summarized under the term of “liquid phase epitaxy” (LPE). The LPE method can deposit thin films having crystalline, high quality coatings and with predefined orientations as the thin films are deposited on the different substrates. Indium-tin oxide (ITO)-based transparent conduction oxide (TCO) thin films have been widely used in the applications of solar cells, flat panel displays [4], and other optoelectronic products due to their excellent conductivity and transparency. Much more interest has been given to ZnO-based thin films such as undoped ZnO thin films [5], Al-doped ZnO (AZO) thin films [6], and F-doped ZnO (FZO) thin films [7] because they have the stability properties even under hydrogen plasma. For that, they are potential candidates for the applications of solar cell technology based on thin-film silicon.

Al-doped ZnO (AZO) are the major materials for ZnO-based thin films, because they present favorable electrical properties and can be investigated as indium-free TCO thin films. In spite of studies on the preparation, characterization, and effect of doping on the properties of ZnO-based thin films, certain effects of either some dopants or preparation procedures still remain unclear. Because Ga is less receptive to oxidation, Ga_2_O_3_-doped ZnO TCO materials have been reported to have a better stable property [8,9]. When Ga_2_O_3_ is served as a dopant, the resistivity of Ga_2_O_3_-doped ZnO thin films is lower than that using other group-III elements [10,11], because Ga has a more compatible covalent bond length with Zn than other group-III elements [12]. TCO thin films deposited on different substrates [13], for example glass and polyimide (PI), are widely used throughout the semiconductor and electronics industries. The polymer substrates are cheaper, lighter, and more flexible as compared with the conventional glass substrates; they could be effectively used in applications such as flexible display and flexible solar cells. For that, the necessity of studying the deposition process of TCO thin films on polymer substrates has been increased, as the polymer substrates are suitable for flat-panel displays (FPDs) and optoelectronics [14,15,16].

Many researchers have reported on the Ga_2_O_3_-doped ZnO thin films in regard to the different doping concentrations of Ga_2_O_3_. In the past, the highly conductive and transparent Ga_2_O_3_-doped ZnO thin films had been deposited at high growth rates by radio frequency magnetron sputtering. In the present paper, a ceramic target of ZnO with 3 wt % Ga_2_O_3_ (ZnO:Ga_2_O_3_ = 97:3 in wt %, abbreviated as GZO) was used to deposit the GZO thin films on the Eagle 173 glass and flexible polyimide (PI) substrates by using the radio frequency (RF) magnetron sputtering process. Then the detail analysis of defects for nano-scale thin films on different substrtaes could be achieved by X-ray photoelectron spectroscopy (XPS) [3]. In this study, the dependences of the structural, electrical, and optical properties of the GZO thin films on different deposition temperatures and substrates were investigated. Also, XPS is a surface-sensitive and a quantitative spectroscopic technique that can be used to measure the variation of electron or x-ray excited from one lower energy layer to one higher energy layer. In order to clarify the mechanism of difference in electrical properties, we would investigate the change of the chemical structures of the GZO thin films deposited on different substrates by XPS.

## 2. Experimental Procedures

In this work, the radio frequency (RF) (13.56 MHz) magnetron sputtering process was used to deposit the GZO thin films. ZnO (97 wt %, 5 N, Admat Inc., Norristown, PA, USA) and Ga_2_O_3_ (3 wt %, 5 N, Admat Inc., Norristown, PA, USA) were mixed, ground, calcined at 1273 K for 2 h, and sintered at 1673 K for 2 h to form the ceramic target with a 2 in diameter. The area of Eagle 173 glass (Corning Inc., New York, NY, USA) and polyimide (abbreviated as PI, Taimide Tech. Inc., Hsinchu County, Taiwan) were used as substrates and their areas were about 33 mm × 33 mm. Before the deposition process was started, the base chamber pressure of the sputtering system was pumped to less than 1 × 10^−6^ Torr, then the deposition parameters were controlled at different pressures and powers. The optimal deposition parameters were a RF power of 50 W and a working pressure of 5 mTorr because the deposited GZO thin films had the flattest surface and the acceptable deposition rate. During the deposition process, only pure argon (99.999%) was introduced in the chamber, the flow rate of argon was 20 sccm. Deposition temperature was also used as a parameter, where GZO thin films were deposited at room temperature (RT, 300 K), 373 K, and 473 K, respectively. The RT was characterized as that the chamber was unheated and the desired temperatures of 373 K and 473 K were achieved by heating of the sample stage using a neon lamp and monitoring the temperature using a thermocouple. Thickness of the GZO thin films was one of the most important parameters to influence the characteristics of the GZO thin films. For this reason, thicknesses of the GZO thin films were measured using field emission scanning electron microscopy (FESEM, JSM-6700, JEOL, Tokyo, Japan). Deposition rate and thickness of the GZO thin films were determined by averaging five data sets obtained by FESEM. After calculating the deposition rate, the thicknesses of all GZO thin films were about 150 ± 10 nm by controlling the deposition time. The roughness (or flatness) was measured using atomic force microscopy (AFM), and the crystalline structure was measured using X-ray diffraction (XRD) patterns with Cu K𝛼 radiation. The electrical properties of the GZO thin films were determined by Hall effect measurement (Ecopia, HMS-3000, Bridge Technol., Chandler Heights, AZ, USA) at room temperature using the Van der Pauw method with four pressed indium balls onto the corners of the samples under a 0.55 T magnetic field. While the optical transmittances of the GZO thin films on glass and PI substrates were measured by using an ultraviolet-visible spectroscopy (UV-Vis) spectrophotometer (Hitachi U3300, Kenichi Sato, Osaka, Japan). The surface chemical composition and bonding of the GZO thin films were analyzed using the X-ray photoemission spectroscopy (XPS, ULVAC-PHI, 5000 Versaprobe, Physical (PHI) Electronics, Chanhassen, MN, USA) after the thin films’ surfaces were pre-cleaning for 3 min by sputtering. The parameters for XPS were monochromatized Al Kα 187.85 eV, presputter setting: 2 kV, sputter area: 2 mm × 2 mm, sputter rate: 12.5 nm/min, sputter time: 0.3 min, and the Ar ions with energy of ~2 keV, respectively.

## 3. Results and Discussion

**Figure 1:**
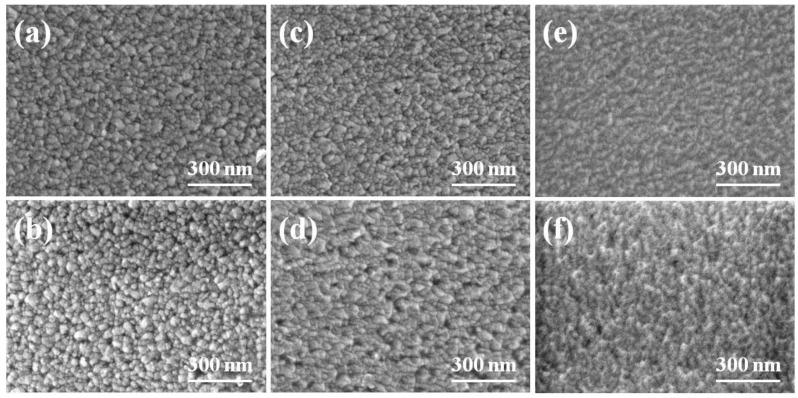
Surface morphologies of the Ga_2_O_3_-doped ZnO (GZO) thin films as a function of substrate and deposition temperature. (**a**) Glass; (**b**) polyimide (PI); (**c**) 373 K-Glass; (**d**) 373 K-PI; (**e**) 473 K-Glass; and (**f**) 473 K-PI, respectively.

Because the GZO thin films were deposited using a sputtering method in a pure Ar atmosphere, we believe that substrate would be the important factor that would influence the properties of the deposited thin films. We used FESEM to observe the surface morphologies of the GZO thin films deposited on different substrates under different deposition temperatures. The morphologies of the GZO thin films as a function of the deposition substrate and temperature are shown in Figure 1, which indicates that as those deposition parameters are changed, the surface morphologies apparently changed as well. As Figure 1a–e show, only nano-scale particles were observed, and the surface morphologies had no apparent change, regardless of the variations of substrates and deposition temperatures. As RT (Figure 1a,b) and 373 K (as Figure 1c,d show) were used as the deposition temperatures, the GZO thin films deposited on glass substrates had less pores than those deposited on PI substrates. As different deposition temperatures were used, the morphology of the GZO thin films exhibited a roughness surface. However, the variations of average crystallization sizes are dependent on the substrate and deposition temperature and they have not easily been calculated from the surface observation. We will illustrate the variations of grain sizes from the XRD patterns and the following equation [17], and the results are also shown in Table 1:

*D* = (0.9λ)/(βcosθ) (1)

where *D* is the size of nano-scale particles; λ is 1.54 Å; β is the full width at half-maximum (FWHM); and θ is the diffraction angle, respectively. As Table 1 shows, the crystallization size increased with the increase of the deposition temperature. This is caused by the fact that as the deposition temperature increases, the surface energy of the GZO thin films during the deposition process increases, and the chance for particles’ growth of the GZO thin films increases, and then their crystallization sizes increase.

**Figure 2:**
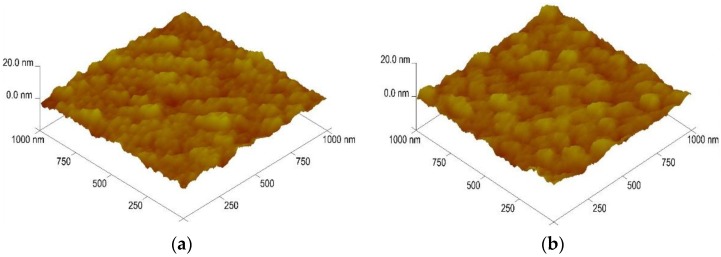
Atomic force microscopy (AFM) analysis of the GZO thin films as a function of substrate at room temperature. (**a**) Glass and (**b**) PI, respectively.

The surface roughness was also calculated as a function of substrate and deposition temperature; the results of the RT-deposited GZO thin films are shown in Figure 2 and the results of all the GZO thin films are also compared in Table 1. The root mean square (RMS) surface roughness of the GZO thin films on glass and PI substrates was measured to be 0.30 nm by AFM. As the deposition temperature was RT, 373 K, and 473 K, the measured values were 1.46 nm, 1.25 nm, and 1.12 nm when the substrate was glass and the measured values were 1.44 nm, 1.22 nm, and 1.10 nm when the substrate was PI, respectively. Apparently, the flatness was improved as the deposition temperature was raised.

**Figure 3:**
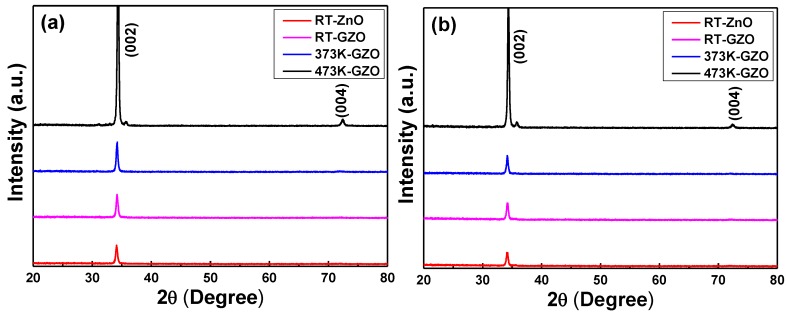
X-ray diffraction (XRD) patterns of the undoped ZnO thin films and GZO thin films as a function of deposition temperature and on different substrates: (**a**) glass and (**b**) PI. a.u.: arbitrary unit.

XRD patterns of the GZO thin films as a function of deposition temperature and substrate were shown in Figure 3, and all the GZO thin films exhibited the (002) peak. XRD patterns of the RT-deposited ZnO thin films are also added in Figure 3 as a reference for the GZO thin films. When glass was used as the substrate, the (002) peaks of the GZO thin films prepared with deposition temperatures of RT, 373 K, and 473 K were situated at 2θ = 34.20°, 34.24°, and 34.36°; when PI was used as the substrate, the (002) peaks of the GZO thin films prepared with deposition temperatures of RT, 373 K, and 473 K were situated at 2θ = 34.14°, 34.18°, and 34.36°, respectively. The (002) peaks of the GZO thin films prepared with a deposition temperature of RT on glass and PI substrates were situated at 2θ = 34.12° and 34.10°. The lattice constant (c) was calculated by using the 2θ values shown in Figure 3 and the results were also compared in Table 1. When glass was used as the substrate, the calculated lattice constant was 0.5240 nm, 0.5234 nm, and 0.5215 nm for deposition temperatures of RT, 373 K, and 473 K; when PI was used as the substrate, the calculated lattice constant was 0.5249 nm, 0.5243 nm, and 0.5215 nm for deposition temperatures of RT, 373 K, and 473 K, respectively. The calculated lattice constants of the ZnO thin films were 0.5252 nm and 0.5255 nm when glass and PI were used as substrates. All the calculated lattice constants c of the GZO thin films in Table 1 being smaller than those of the ZnO thin films is significant, because the radius of Ga^3+^ ions (62 pm) is smaller than that of Zn^2+^ ions (72 pm). Table 1 also shows an important result: the lattice constant of the GZO thin films decreases with the increase of the deposition temperature, independent of the substrate. The lattice parameters of TCO-based thin films usually depend on the concentration of foreign atoms, defects, external strain, and the difference of their ionic radii with respect to the substituted matrix ions. Those results suggest that as the deposition temperature increases, more Ga^3+^ ions will substitute the sites of Zn^2+^ ions and the lattice constant decreases. The study of Li *et al.* suggested that the oxygen vacancies might reduce the lattice parameters, at least the c value [18].

As Figure 3 shows, the full width at half maximum (FWHM) values of the (002) peak of the GZO thin films deposited on different substrates were calculated, and the results are compared in Table 1. The FWHM values of the (002) peak for GZO thin films deposited on glass substrates were 0.407, 0.336, and 0.265 for substrate temperatures of RT, 373 K, and 473 K; the FWHM values of the (002) peak for GZO thin films deposited on PI substrates were 0.412, 0.360, and 0.292 for substrate temperatures of RT, 373 K, and 473 K, respectively. These results suggest that the GZO thin films deposited at higher temperatures have a better crystalline structure.

**Figure 4:**
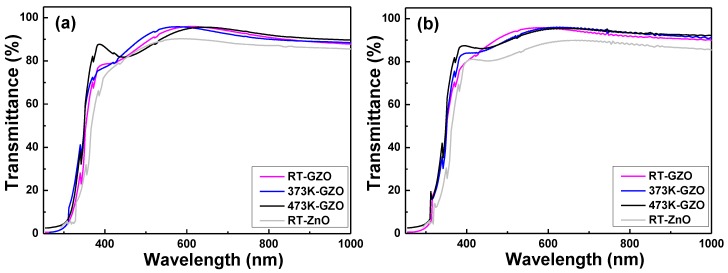
Transmittance spectra of the undoped ZnO thin films and GZO thin films deposited as a function of deposition temperature and different substrates: (**a**) glass and (**b**) PI.

Figure 4 shows the transmission spectra of the GZO thin films plotted against the different deposition temperature and on different substrates; the measured wavelength was in the region of 300–1000 nm. The transmission spectrum of the RT-deposited ZnO thin films is also added in Figure 4 as a reference for the GZO thin films. For the GZO thin films, the absorption edges were blue-shifted and the transmittance ratios were higher as compared with that of the RT-deposited ZnO thin films. As Figure 4 shows, the maximum transmittance ratios of all the GZO thin films in the range of visible light (400–700 nm) were more than 95%, regardless of deposition temperature and substrate. We know that many factors will affect the transmission spectrum of the GZO thin films. The increase of the transmission ratio in the optical band is caused by the increase in carrier density, because a higher deposition temperature can cause the GZO thin films to have fewer defects. The higher deposition temperature could even cause the GZO thin films to have better crystallization; the average and maximum transmittance ratios of all the GZO thin films in the region of visible light had no apparent variation as the deposition temperature increased. The reason is that the optical band gap (*Eg*) of the GZO thin films is in the region of ultraviolet light, thus the optical energy in the region of visible light cannot excite the electrons, and then the GZO thin films in the range of visible light will have high transmittance ratios. As Figure 4 shows, the average transmittance ratios of all the GZO thin films in the range of visible light were about 90.9%–92.8% and in the range of near-infrared light (700–1500 nm) they were about 84.8%–88.9%, respectively. The detailed optical properties of the GZO thin films deposited on different substrates and at different temperatures and ZnO thin films deposited on different substrates are compared in Table 2.

In the transmission spectra of all the GZO thin films, a greater sharpness was noticeable in the curves of the absorption edges, and the optical band edges had no apparent shift as the deposition temperature increased. In the past, determination of the optical band gap (*Eg*) was often necessary to develop the electronic band structure of a thin-film material. However, using extrapolation methods, the *Eg* values of the GZO thin films can be determined from the absorption edge for direct interband transition, which can be calculated using the relation in Equation (3) as follows:

αhν = C × (hν − *Eg*)^1/2^ (2)

where C is the constant for the direct transition, h is Planck’s constant, and ν is the frequency of the incident photon [19]; α is the optical absorption coefficient, which is calculated using Lambert’s law as follows:

α = ln((1/*T*)/*h*) (3)

where *T* and *h* are the thin film’s transmittance ratio and thickness.

**Figure 5:**
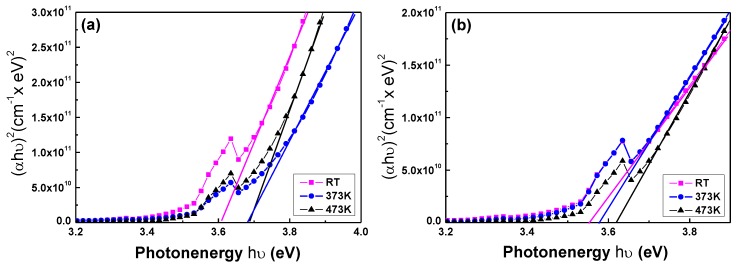
The (αhν)^2^ *vs.* hν*-Eg* plots of the GZO thin films deposited as a function of deposition temperature and on different substrates: (**a**) glass and (**b**) PI.

Figure 5 plots (αhν) against (hν) (energy) in accordance with Equation (3), and the *Eg* values can be found by extrapolating a straight line at (αhν) = 0. The calculated *Eg* values of the GZO thin films as a function of the deposition temperature and on different substrates are also shown in Figure 5. The linear dependence of (αhν) on (hν) indicates that the GZO thin films are direct transition-type semiconductors. As the deposition temperature increased from RT to 473 K and glass was used as the substrate, the *Eg* values increased from 3.612 eV to 3.677 eV and the wavelength of the absorption edge decreased from about 360 nm (transmittance ratio over 60%) to about 352 nm, where the wavelengths of the absorption edge were blue-shifted as compared with those investigated by Roth *et al.* [20]. As the deposition temperature increased from RT to 373 K and PI was used as the substrate, the *Eg* values increased from 3.551 eV to 3.622 eV and the wavelength of the absorption edge decreased from 356 nm to 350 nm. As compared with the results deposited at room temperature by Gong *et al.*, the deposited GZO thin films had *Eg* values changed from 3.42 eV to 3.50 eV [21] and had an absorption edge at the wavelength of about 400 nm. Apparently, for all the GZO thin films investigated in this study the *Eg* values were larger than those investigated by Gong *et al.* The *Eg* values are 3.320 eV (glass) and 3.317 eV (PI) in this study (*Eg* is about 3.24 eV in [22]). As compared with those of the RT-deposited ZnO thin films, the reason for the blue-shift in the absorption edge of the GZO thin films (Figure 3) is caused by the increase of the *Eg* values. The detailed *Eg* values of the GZO thin films deposited on different substrates and at different temperatures and ZnO thin films deposited on different substrates are also compared in Table 2.

Two competing phenomena are generally agreed to be dominant in affecting the absorption edge in heavily doped semiconductors. The first is the well-known Burstein-Moss band-filling effect which negatively shifts the measured band-edge energy with the decrease of the carrier concentration; the second phenomenon that affects the optical absorption edge is the increase of donor density, which will cause a change in the nature and strength of the interaction potentials between donors and the host crystal. The carrier concentration, which will be shown in Figure 5a, is higher than that investigated by Roth *et al.* [20], and the wavelength of the absorption edge can be shifted to a lower value of about 350 nm. The *Eg* value of Ga_2_O_3_ is about 4.9 eV [23] and the *Eg* value of ZnO is about 3.24 eV. We believe that the *Eg* values of all the deposited GZO thin films higher than those of the ZnO thin films are caused by the addition of Ga_2_O_3_ into the ZnO.

**Figure 6:**
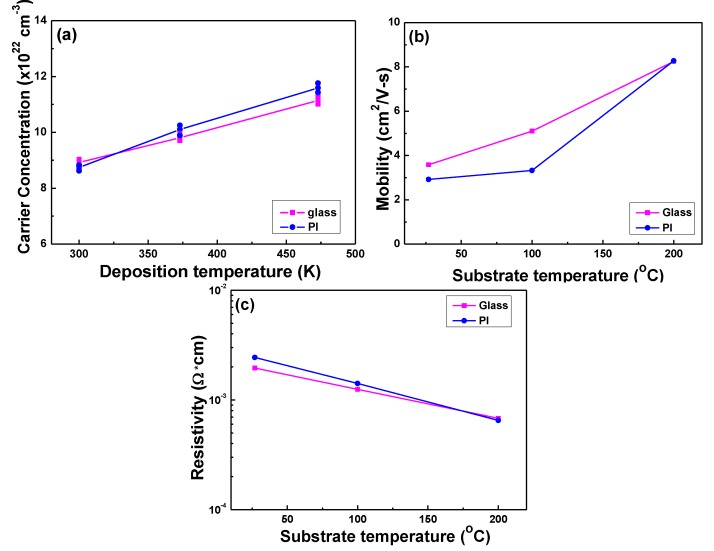
Variations of the (**a**) Hall mobility; (**b**) carrier concentration; and (**c**) resistivity of the GZO thin films as a function of used substrates and deposition temperature.

The variations of the carrier concentration, carrier mobility, and resistivity of the GZO thin films at different deposition temperatures on glass and PI substrates are compared Figure 6. Figure 6a shows that the carrier concentration of the GZO thin films increased with the raising deposition temperature. When the GZO thin films are deposited on glass or PI substrates by using the RF sputtering process, many defects and pores will exist and inhibit the electrons’ movement. Using a higher deposition temperature during the deposition process can lead to an enhancement of the thin films’ densification and crystallization; the increase in the diffraction intensity of the (002) peak, as shown in Figure 3, has proven those results. That can decrease the numbers of defects and pores in the GZO thin films and can decrease the inhibition of the barrier electron transportation [24]. Also, as the deposition temperature was raised from RT to 473 K, the carrier (electron) concentration increased from 8.92 × 10^20^ to 11.1 × 10^20^ cm^−3^ for glass substrates and from 8.74 × 10^20^ to 11.6 × 10^20^ cm^−3^ for PI substrates. Figure 6b shows that the carrier mobility of the GZO thin films increased with the raising deposition temperature and was almost independent of the used substrates. Figure 6b also shows that as the same deposition temperature was used, the carrier mobility of the GZO thin films on glass substrates was higher than that of the GZO thin films on PI substrates. As the deposition temperature was raised from RT to 473 K, as Figure 6b shows, the carrier mobility increased from 3.58 to 8.25 cm^2^/V-s for glass substrates and increased from 2.92 to 8.28 cm^2^/V-s for PI substrates. The improvement in electron mobility could be attributed to the re-crystallization of the GZO thin films that takes place during the deposition process with the increase of the deposition temperature.

Figure 6c shows the dependence of the resistivity of the GZO thin films on deposition temperature and the used substrate. Resistivity of the GZO thin films is proportional to the reciprocal value of the product of the carrier concentration N and the mobility μ:

ρ = 1/(N × e × μ)
(4)

As Equation (4) shows, both the carrier concentration and the carrier mobility contribute to the resistivity. The results in Figure 6c indicate that the resistivity decreased with the increase of the deposition temperature. Thus, the increases in carrier concentration and electron mobility should be taken into account when we analyze the decrease in resistivity of the GZO thin films. Those results in Figure 6a,b suggest that reduction in resistivity also comes from an increase in electron mobility in addition to the increase of the electron concentration. In this study, the minimum resistivity of the GZO thin films at a deposition temperature of 473 K on different substrates is mainly influenced by both the carrier concentration and the carrier mobility being at their maximum. As the deposition temperature was raised from RT to 473 K, the resistivity decreased from 1.95 × 10^−3^ to 0.680 × 10^−3^ Ω·cm for glass substrates and from 2.44 × 10^−3^ to 0.651 × 10^−3^ Ω·cm for PI substrates, respectively.

**Figure 7:**
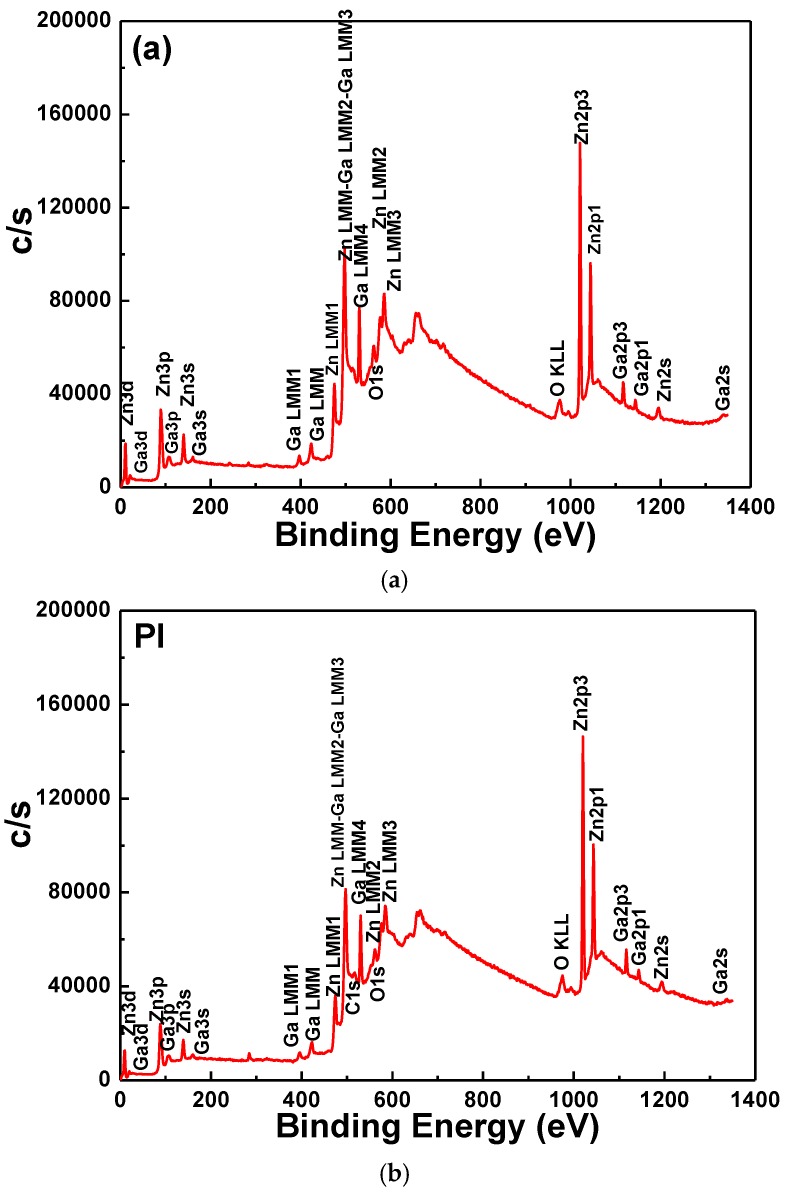
Typical widescan spectrum of the GZO thin films as a function of used substrates. (**a**): glass; (**b**): PI.

Figure 7 shows a typical widescan spectrum of the GZO thin films as a function of the used substrates. The photoelectron peaks of the main elements, Zn, O, and Ga, and Auger Zn and Ga *LMM* (electron excited from L layer to M layer) and O *KLL* (electron excited from K layer to L layer) peaks were obtained. As Figure 7 shows, some small peaks with intensities lower than each main photoelectron peak were also observed.

**Figure 8:**
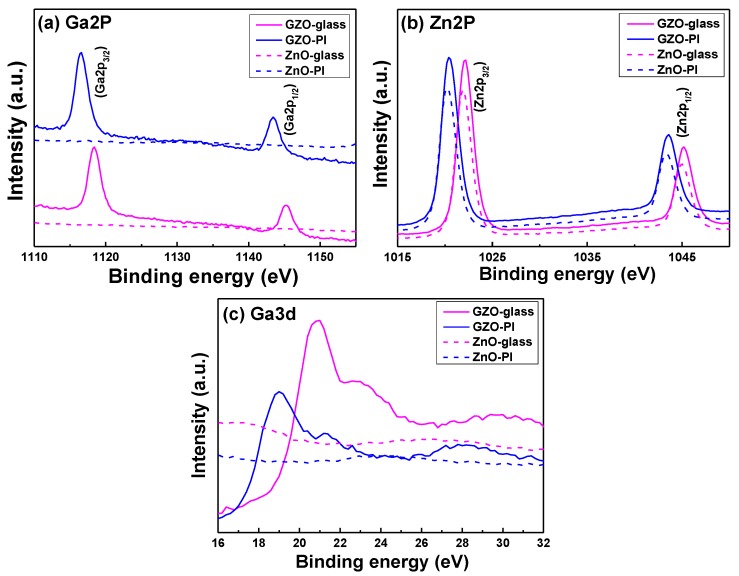
Binding energy spectra of (**a**) Ga2p_3/2_ and Ga2p_1/2_ peaks; (**b**) Zn2p_3/2_ and Zn2p_1/2_ peaks; and (**c**) Ga3d peak for the undoped ZnO thin films and GZO thin films on different substrates.

To clarify the mechanism of improvement in electrical properties, the chemical structures of the GZO thin films were investigated by XPS. The XPS spectra for the Ga2p_1/2_, Ga2p_3/2_, Zn2p_1/2_, Zn2p_3/2_, and Ga3d peaks of the GZO thin films are shown in Figure 8 as a function of the used substrates, and the values are compared in Table 3; those thin films were deposited at RT. The XPS spectra for the Ga2p_1/2_, Ga2p_3/2_, Zn2p_1/2_, Zn2p_3/2_, and Ga3d peaks of the ZnO thin films are also shown in Figure 8 for comparison. When glass was used as the substrate, the binding energy of each constituent element was positioned at 1145.22 eV (Ga2p_1/2_), 1118.36 eV (Ga2p_3/2_), 1045.23 eV (Zn2p_1/2_), 1022.10 eV (Zn2p_3/2_), and 20.72 eV (Ga3d), respectively, as calibrated to 285.43 eV (C1s); When PI was used as the substrate, the binding energy of each constituent element was positioned at 1143.35 eV (Ga2p_1/2_), 1116.52 eV (Ga2p_3/2_), 1043.64 eV (Zn2p_1/2_), 1020.47 eV (Zn2p_3/2_), and 19.06 eV (Ga3d), respectively. We know the binding energies of Ga_2_O_3_ and Ga metal are 1117.4 ± 0.5 eV and 1116.5 ± 0.2 eV. As Figure 8a shows, no apparent peak could be observed in the XPS spectra of the Ga3d peak of the ZnO thin films. For that, the results in Figure 8a suggest that the binding state of Ga_2_O_3_ will dominate the GZO thin films on glass and the binding state of Ga will dominate the GZO thin films on PI.

As Figure 8b shows, two similar peaks were observed in the XPS spectra of the ZnO and GZO thin films. The binding energy peak at 1022.40 ± 0.10 eV could be due to Zn2p_3/2_ in the ZnO_1-*x*_ structure and the binding energy peak at 1021.1 ± 0.40 eV was due to metallic zinc [25]. The results in Figure 8b suggest that the binding state of ZnO_1-*x*_ will dominate the GZO thin films on glass and the binding state of Zn will dominate the GZO thin films on PI. The binding energy of the Ga3d peak for Ga_2_O_3_ (Ga^3+^) is 20.7 eV; the binding energies of the Ga3d peak for Ga_2_O (Ga^1+^) or GaO (Ga^2+^) are about 20.7 eV; and the binding energy of the Ga3d peak for Ga metal is 18.6 ± 0.3 eV, respectively. Figure 8c shows the binding energy of the Ga3d peak of the GZO thin films on different substrates. When glass was used as the substrate, the binding energy of the Ga3d peak was about 20.7 eV, which suggests the binding energy of the Ga3d peak is Ga_2_O_3_ (Ga^3+^); when PI was used as the substrate, the binding energy of the Ga3d peak was about 18.6 ± 0.3 eV, which suggests the binding energy of the Ga3d peak is the Ga metal. No apparent peaks could be observed in the XPS spectra of the Ga3d peak of the ZnO thin films. For that, the binding energies in Figure 8c reveal the same results as those of Figure 8a. The results in Figure 8 showed significant variations in the core binding energies (~1.55 eV) as we varied the substrates. The change can be related to the difference between the intrinsic properties of the deposition materials as well as to the differences in the interaction with the substrates [26].

**Figure 9:**
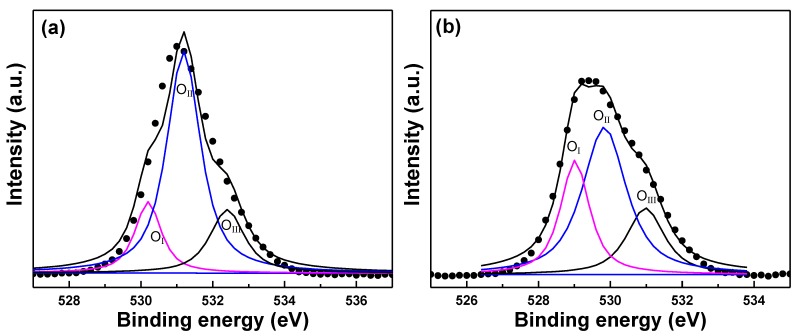
Binding energy spectra of O_1__s_ peaks for GZO thin films on different substrates: (**a**) glass and (**b**) PI.

Figure 9a shows the XPS spectra for the Gaussian-resolved components of O_1s_ of the GZO thin films on glass substrates; those thin films were deposited at RT. The bonding energy components were centered at 531.17 eV and 529.35 eV for glass and PI substrates, respectively. As shown in Figure 9a, two shoulders were visible on the low-energy side and high-energy side of the GZO thin films, indicating that at least three different bonds existed. For GZO thin films deposited on glass substrates, the bonding state of the O_1s_ spectrum was resolved into three components centered at 530.21 eV, 531.18 eV, and 532.39 eV for the O_I_, O_II_, and O_III_ peaks, respectively. As shown in Figure 9b, only one shoulder was visible on the high-energy side of the GZO thin films, and we also found that three different bonds existed. For GZO thin films deposited on PI substrates, the bonding state of the O_1s_ spectrum was resolved into three components centered at 529.03 eV, 529.83 eV, and 531.02 eV for the O_I_, O_II_, and O_III_ peaks, respectively. Table 4 shows the area ratios of the O_I_, O_II_, and O_III_ peaks; the area beneath the peaks is clearly different for these two substrates.

For GZO thin films deposited on glass (PI) substrates, the low bonding energy component of the O_I_ peak located at 530.21 ± 0.15 eV (529.03 ± 0.15 eV) is attributed to O^2^^−^ ions on the wurtzite structure of the hexagonal Zn^2+^ ion array, surrounded by Zn atoms with their full complement of nearest-neighbor O^2^^−^ ions [25,27]. The second binding energy component of the O_II_ peak located at 531.18 ± 0.15 eV (529.83 ± 0.15 eV) is associated with O^2^^−^ ions in the oxygen vacancies within the matrix of ZnO_1__−_*_x_* [28], which is also known as V_O_-like bonding. The highest bonding energy component of the O_III_ peak located at 532.39 ± 0.15 eV (531.02 ± 0.15 eV) implies the presence of hydrated oxide species on the film surface [29]. As Figure 9a shows, when glass was used as the substrate and the deposition was RT, the area ratio of the O_II_ peak was larger than those of the O_I_ and O_III_ peaks. These results suggest that there are more oxygen vacancies existing in GZO thin films to cause the increase in the carrier concentration. When PI was used as the substrate, because the PI substrates had less surface energy, that would cause the GZO thin films to have poor adhesive force, and the GZO thin films on PI substrates would have poor crystallization, as Figure 3 shows. The area ratio of the O_II_ peak apparently decreased and the area ratios of the O_I_ and O_III_ peaks increased. These results are caused by the decrease of oxygen vacancies and the increase of oxygen absorption, which will degenerate the electrical properties of the GZO thin films, as Figure 6 shows. These results suggest that even with the same deposition parameters, the GZO thin films deposited on different substrates will have different results.

## 4. Conclusions

In this study, the high-transmittance nano-scale GZO thin films were successfully deposited on glass and PI substrates by using the RF sputtering process. For all GZO thin films, the transmittance ratios of the GZO thin films at 400–700 nm were more than 94% regardless of deposition temperature and substrate. As the deposition temperature increased from RT to 473 K and glass (PI) was used as the substrate, the FWHM values of the (002) peak for GZO thin films deposited on glass (PI) substrates were 0.407 (0.412), 0.336 (0.360), and 0.265 (0.292) for substrate temperatures of RT, 373 K, and 473 K; the *Eg* values increased from 3.612 (3.551) eV to 3.677 (3.622) eV and the wavelength of the absorption edge decreased from about 360 (356) nm to about 352 (350) nm, respectively. From the binding energy spectra of Ga2p_3/2_ and Ga2p_1/2_ peaks, Zn2p_3/2_ and Zn2p_1/2_ peaks, the Ga3d peak, and O_1s_ peaks, we had proved that GZO thin films on different substrates had different chemical and electronic states, and thus different electrical properties.

## Figures and Tables

**Table 1:** The 2θ value of the (002) peak, crystallite size (*D*), lattice constant (c), the full width at half-maximum (FWHM) value, and roughness of the Ga_2_O_3_-doped ZnO (GZO) thin films deposited on different substrates and at different temperatures and ZnO deposited on different substrates. RT: room temperature; PI: polyimide.

Substrates	2θ (°)	Crystallite Size (nm)	c (nm)	FWHM (°)	Roughness (nm)
RT glass	34.20	31.34	0.5240	0.407	1.46
RT PI	34.14	28.12	0.5249	0.412	1.44
373 K glass	34.24	24.71	0.5234	0.336	1.25
373 K PI	34.18	23.08	0.5243	0.360	1.22
473 K glass	34.36	20.42	0.5216	0.265	1.12
473 K PI	34.36	20.18	0.5216	0.296	1.10
RT ZnO glass	34.12	23.42	0.5252	0.382	1.24
RT ZnO PI	34.10	24.45	0.5255	0.406	1.26

**Table 2:** Optical properties and the optical band gap (*Eg*) values of the GZO thin films deposited on different substrates and at different temperatures and ZnO thin films deposited on different substrates.

Substrates	Transmittance Ratio-Visible	Transmittance Ratio-Infrared	Maximum Transmittance Ratio	Absorption Edge (nm)	*Eg* Value (eV)
RT glass	90.9%	85.0%	95.9%	360	3.612
373 K glass	91.6%	84.8%	95.8%	354	3.674
473 K glass	90.5%	87.2%	95.6%	352	3.677
RT PI	92.8%	86.8%	95.8%	356	3.551
373 K PI	92.4%	87.0%	96.1%	354	3.580
473 K PI	92.4%	88.9%	95.5%	350	3.622
RT ZnO glass	87.1%	82.6%	90.2%	370	3.320
RT ZnO PI	86.1%	82.1%	89.9%	368	3.317

**Table 3:** Comparison of binding energies of Ga2p_3/2_, Ga2p_1/2_, Zn2p_3/2_, Zn2p_1/2_, and Ga3d peaks for GZO thin films on different substrates.

Title	Glass	PI
Ga2P_1/2_ Binding energy (eV)	1145.22	1143.35
Ga2P_3/2_ Binding energy (eV)	1118.36	1116.52
Zn2p_1/2_ Binding energy (eV)	1045.23	1043.64
Zn2p_3/2_ Binding energy (eV)	1022.10	1020.47
Ga3d Binding energy (eV)	20.72	19.06

**Table 4:** Comparison of area ratios of O_I_, O_II_, and O_III_ peaks for GZO thin films on different substrates.

Title	Glass-RT	PI-RT
O_I_ peak	14.1%	20.3%
O_II_ peak	81.1%	52.6%
O_III_ peak	4.8%	27.0%
